# Supplementary material for: The causal relationship between the human gut microbiota and pyogenic arthritis: a Mendelian randomization study
Source: Front Cell Infect Microbiol. 2024 Nov 26;14:1452480. doi: 10.3389/fcimb.2024.1452480 (PMC11629706; doi:10.3389/fcimb.2024.1452480)
Supplement: Supplementary file 1 [file DataSheet1.zip › mendelian test/ebi-a-GCST90027798.csv_forest.pdf]

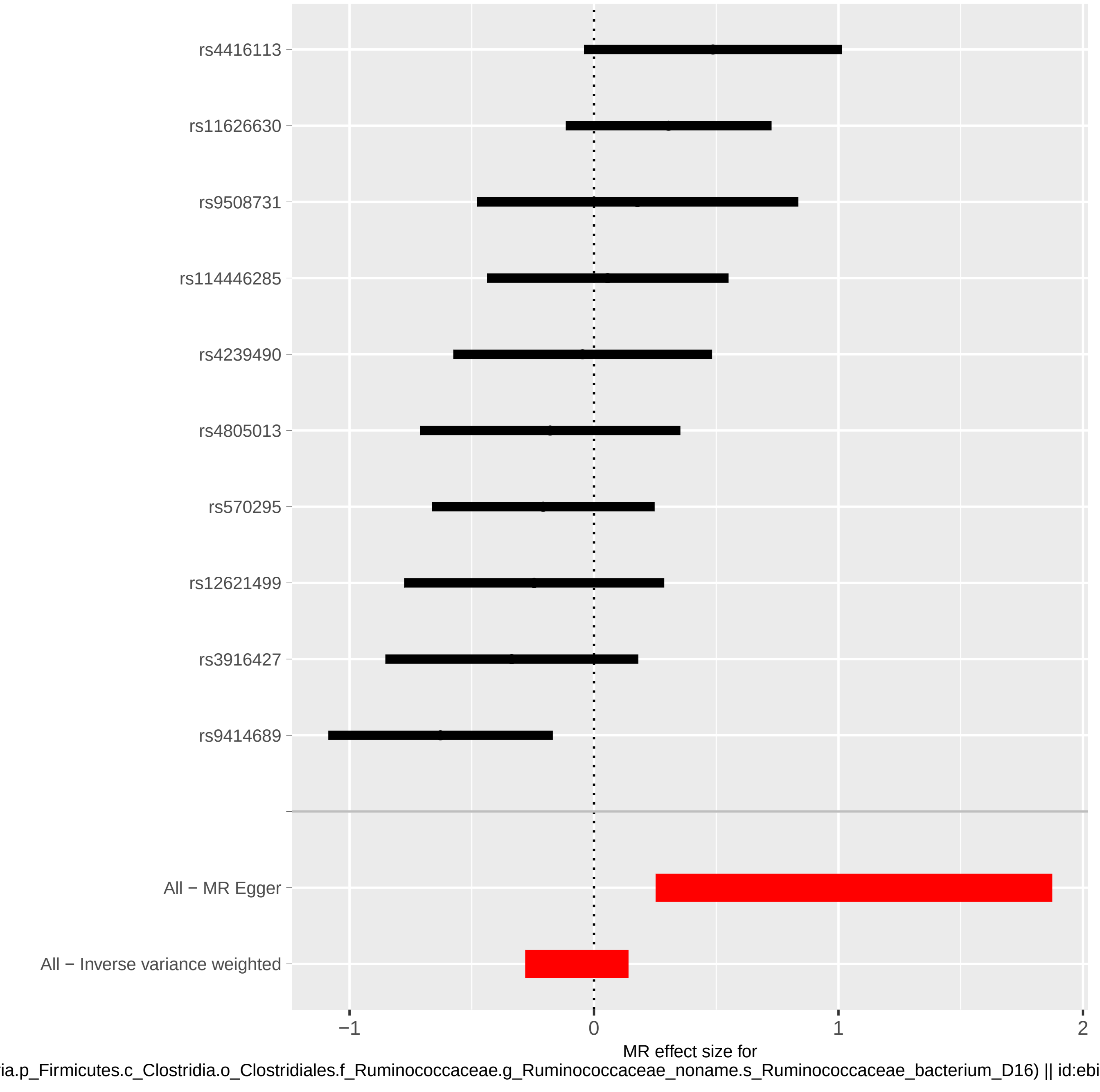
rs4416113
rs11626630
rs9508731
rs114446285
rs4239490
rs4805013
rs570295
rs12621499
rs3916427
rs9414689
All − MR Egger
All − Inverse variance weighted
−1
0
1
2
MR effect size for
ia.p_Firmicutes.c_Clostridia.o_Clostridiales.f_Ruminococcaceae.g_Ruminococcaceae_noname.s_Ruminococcaceae_bacterium_D16) || id:ebi
